# Curcumin Alleviates Matrix Metalloproteinase-3 and -9 Activities during Eradication of *Helicobacter pylori* Infection in Cultured Cells and Mice

**DOI:** 10.1371/journal.pone.0016306

**Published:** 2011-01-21

**Authors:** Parag Kundu, Ronita De, Ipsita Pal, Asish K. Mukhopadhyay, Dhira Rani Saha, Snehasikta Swarnakar

**Affiliations:** 1 Indian Institute of Chemical Biology, Kolkata, India; 2 National Institute of Cholera and Enteric Diseases, Kolkata, India; Louisiana State University, United States

## Abstract

Current therapy-regimens against *Helicobacter pylori* (Hp) infections have considerable failure rates and adverse side effects that urge the quest for an effective alternative therapy. We have shown that curcumin is capable of eradicating Hp-infection in mice. Here we examine the mechanism by which curcumin protects Hp infection in cultured cells and mice. Since, MMP-3 and -9 are inflammatory molecules associated to the pathogenesis of Hp-infection, we investigated the role of curcumin on inflammatory MMPs as well as proinflammatory molecules. Curcumin dose dependently suppressed MMP-3 and -9 expression in Hp infected human gastric epithelial (AGS) cells. Consistently, Hp-eradication by curcumin-therapy involved significant downregulation of MMP-3 and -9 activities and expression in both cytotoxic associated gene (*cag*)^+ve^ and *cag* ^-ve^ Hp*-*infected mouse gastric tissues. Moreover, we demonstrate that the conventional triple therapy (TT) alleviated MMP-3 and -9 activities less efficiently than curcumin and curcumin's action on MMPs was linked to decreased pro-inflammatory molecules and activator protein-1 activation in Hp-infected gastric tissues. Although both curcumin and TT were associated with MMP-3 and -9 downregulation during Hp-eradication, but unlike TT, curcumin enhanced peroxisome proliferator-activated receptor-γ and inhibitor of kappa B-α. These data indicate that curcumin-mediated healing of Hp-infection involves regulation of MMP-3 and -9 activities.

## Introduction

*Helicobacter pylori* (Hp) has been implicated in the pathogenesis of most important gastroduodenal diseases, such as gastritis, peptic ulcer, gastric carcinoma and has been defined as a Class I carcinogen [1]. Hp can be subclassified into ‘cag’ pathogenicity island positive (*cag*)^+ve^ and negative (*cag*)^-ve^ strains based on the presence or absence of *cag*PAI, a 40-kb genome fragment containing 31 genes [2]. There is enormous heterogeneity in the consequences of Hp-infections, however more severe disease manifestations have been attributed to infection by *cag* ^+ve^ isolates [3].

Currently, the most preferred Hp eradication therapy (triple-therapy) employ, one proton pump inhibitor and two antibiotics [4]. However, such multiple therapy regimens have not been very successful in clinical practice, since the overuse or rather misuse of antibacterial agents have resulted in the emergence of antibiotic-resistant strains which is the prime cause of treatment failure apart from potential side effects [4], [5]. Increasing complications in the conventional triple-therapy (TT) stimulate an urgent need to develop new non-antibiotic antibacterial agents against Hp-infection that are safe, highly effective and have specific cellular targets.

Several studies have demonstrated that Hp-infection induces the secretion of matrix metalloproteinases (MMPs) from a variety of gastric cells *in vivo* as well as in cultured cells, which in turn contribute to the pathogenesis of gastric ulcer and gastric cancer [6]–[10]. Gastric epithelial cells appear to be the major source of MMPs in Hp infected gastric tissues [11]. MMPs are a family of diverse zinc dependant endopeptidases that have broad substrate specificity and play a crucial role in various physiological processes including tissue remodeling, organ development, wound repair and inflammatory processes [12]–[14]. Among them, gelatinases, (MMP-2 and MMP-9) and stromelysin-1 (MMP-3) collectively cleave gelatins (types I and V), collagens (type IV, V, VII, IX and X), elastin, fibronectin, laminin and proteoglycan core proteins [12]. The activities of MMPs are regulated by their inhibitors (TIMPs), while their gene expressions are modulated by cytokines, growth factors, tumour promoters and transcription factors including nuclear factor (NF)-κB and activator protein (AP)-1 [15]. The dynamic equilibrium between MMPs and TIMPs is a critical factor for diverse cellular functions including cellular proliferation, migration, adhesion and apoptosis [16].

Curcumin (diferuloylmethane) the principle yellow pigment present in the rhizome of turmeric (*Curcuma longa*), has a wide array of pharmacological and biological activities. Studies on the safety of *C*. *longa* and its derivatives in different animal models [17], have shown that even at high doses turmeric is non-toxic to laboratory animals. Apart from its antioxidant, anti-inflammatory, anti-infectious and anti-carcinogenic properties, curcumin has been shown to target several molecules like growth factors, transcription factors, cytokines and enzymes including MMPs, that are involved in the etiology of diverse diseases [9], [18]-[20]. Recently we and others have shown that curcumin possesses anti-Hp potential *in vitro*, and also protective effect on Hp-infection in mice [18], [19], [21]–[23].

We primarily tested the effect of curcumin on the activity and protein levels of MMP-3 and -9 during protection against Hp-infection in cultured cells and mice. Secondly, we compared the efficiency of curcumin with conventional TT in stabilizing the altered balance between MMPs and TIMPs that is critical for tissue repair following Hp eradication. Finally, we sought to determine the molecular mechanisms underlying the regulation of MMP-3 and -9 by curcumin and TT against Hp infection in mice. To our knowledge, this study is the first to document the biochemical changes at the level of MMPs brought about by curcumin as well as TT treatments in Hp infected mice.

## Results

### Curcumin dose dependently suppresses MMP-3 and -9 expressions during protection against Hp-infected cultured cells

**Figure 1:**
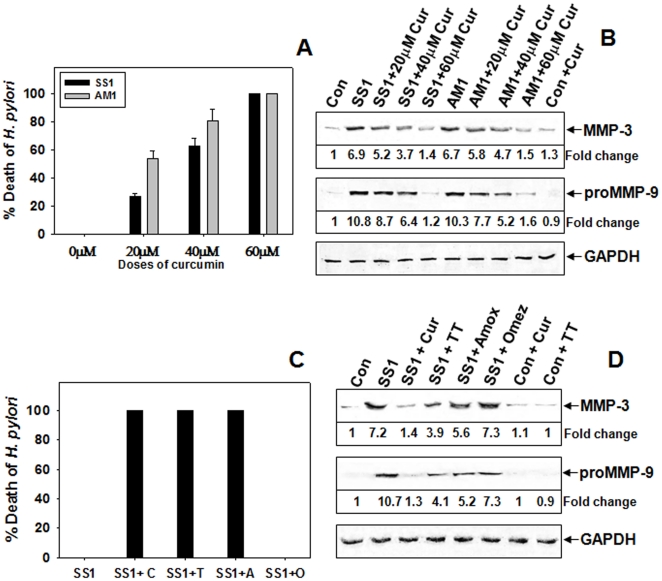
Dose dependent downregulation of secreted MMPs by curcumin on cag^+ve^ and cag^-ve^ Hp-infected AGS cells. AGS cells cocultured with SS1 (cag^+ve^) and AM1 (cag^-ve^) Hp strains were pretreated with 20 µM, 40 µM, 60 µM doses of curcumin and the effect of increasing doses of curcumin on Hp viability during coculture of Hp and AGS cells for 24 h were represented in the histogram (**A**). The MMP-3 and -9 levels in the conditioned media after 24 h were analyzed. Western blots (**B**) were performed using equal volumes of concentrated media followed by probing with polyclonal anti-MMP-3, and -GAPDH and monoclonal anti-MMP-9 antibodies. AGS cells alone were considered as control. AGS cells cocultured with SS1 Hp strain were pretreated with curcumin or TT or only-antibiotics or omeprazole and the MMP levels in the conditioned media after 24 h were assessed. Histographic representation of Hp-viability (**C**) after 24 h of coculture against various treatments. Concentrated conditioned media from each group of treatment were analyzed by Western blot (**D**) for MMP-3 and MMP-9 secretion level by probing with polyclonal anti-MMP-3, and GAPDH and, monoclonal anti-MMP-9 antibodies. Fold changes of protein were calculated from the above blots and two other representative blots from independent experiments.

**Figure 2:**
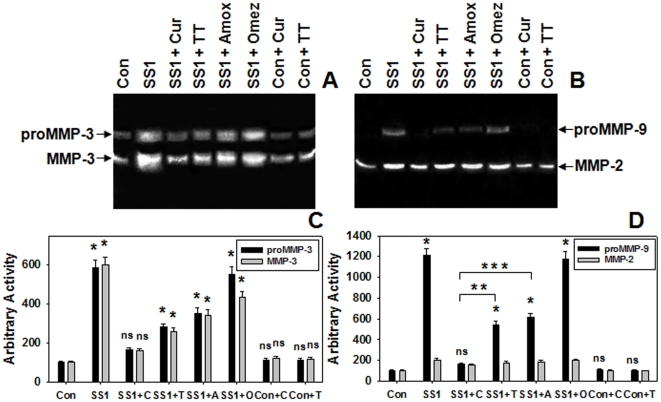
Comparative regulation of secreted MMP-3 and -9 in Hp-infected AGS cells by curcumin and TT. AGS cells cocultured with SS1 Hp strain were pretreated with curcumin or TT or only-antibiotics or omeprazole and the MMP levels in the conditioned media after 24 h were assessed. Concentrated conditioned media were analyzed by casein (**A**) and gelatin (**B**) zymography. Histographic representation of caseinolytic (**C**) and gelatinolytic (**D**) activities from the above zymogram and two other representative zymograms from independent experiments. Error bars = ±SEM. *, p<0.001; ns, nonsignificant vs. AGS cells alone. **, p = 0.0002 and ***, p = 0.0003 vs curcumin treated Hp infected AGS cells.

Since MMP-3 and -9 plays a pivotal role in degrading majority of gastric ECM proteins during Hp-induced pathogenesis, we investigated the effects of curcumin on the levels MMP-3 and -9 in Hp-infected cultured cells. Initially, the antibacterial activity of curcumin against cag^+ve^ SSI and cag^-ve^ AM1 Hp strain viability was tested. AGS cells cocultured with SS1 and AM1 Hp-strains were pretreated with varying doses of curcumin and the MMP levels in the conditioned media after 24 hours was analyzed. The effect of curcumin on the viability test of Hp in infected cells revealed that both SS1 and AM1 bacterial viability declined with increasing doses of curcumin and they were completely eliminated at 60 µM concentration after 24 hours (Figure 1A). Since curcumin was most effective at 60 µM dose, we used this dose in further cell culture experiments. Figure 1B depicts that curcumin dose dependently suppressed the increased secretion of MMP-3 and -9 in SS1 or AM1 strain infected cells which declined to almost control level at 60 µM curcumin dose. Presently omeprazole based triple-therapy (TT) is used to treat Hp infection. The efficacy of curcumin compared to TT, antibiotic alone and omeprazole was tested in SS1 strain infected AGS cells. As expected only omeprazole did not inhibit the growth of Hp in infected AGS cells while only antibiotic, TT and curcumin completely inhibited the bacterial growth (Figure 1C). Consequently, the secreted MMP-3 and -9 was inhibited 90% by curcumin while 50% by TT and antibiotic alone (Figure 1D). The negative regulation of curcumin on the secretion of MMP-3 and -9 was also much pronounced than the other treatments in AGS cells cocultured with SS1 as evident in casein and gelatine zymography (Figure 2). Overall, cucumin's action on MMP-3 and -9 was significant while the effect of TT and only antibiotics was moderate.

### Curcumin dose dependently suppresses MMP-3 and-9 during eradication of Hp from infected mice

**Figure 3:**
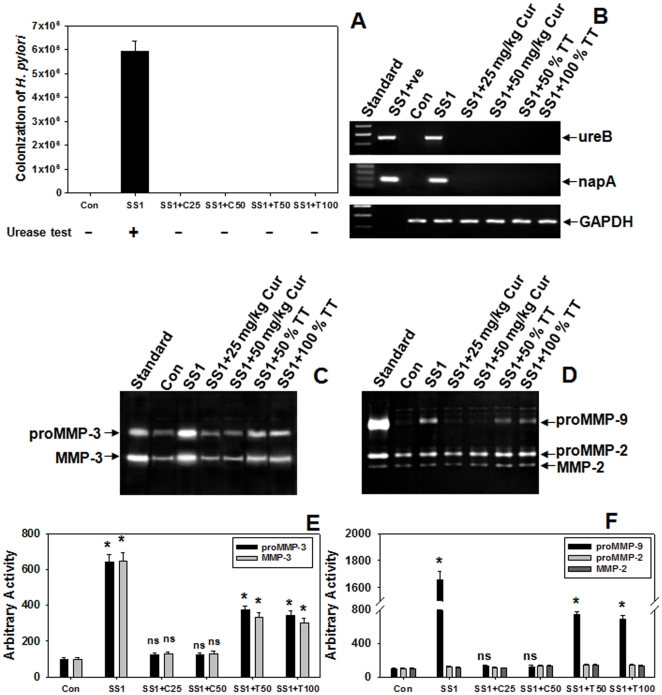
Effect of varying doses of curcumin and TT on Hp-viability and secreted-MMPs in Hp-infected mice. Two weeks SS1 infected mice groups were treated with increasing doses of curcumin (25 mg/kg or 50 mg/kg) or TT (50% or 100% of human dose) for 7-days. Histographic representation of colonization of SS1 strain, obtained by quantitative culture, in mouse and the effect of varying doses of curcumin or TT thereon. Results of urease-test for the respective samples were stated (**A**). Test for the presence of Hp-specific genes *UreB* and *NapA* and mouse-specific *GAPDH* gene in the DNA isolated from the gastric tissues of mice (**B**). The activities of MMP-3 and gelatinases in the PBS extracts of respective mouse gastric tissues were analyzed by casein (**C**) and gelatin (**D**) zymography respectively. Histographic representations of caseinolytic (**E**) and gelatinolytic (**F**) activities from the above zymograms and three other representative zymograms from independent experiments. Error bars = ±SEM. *, p<0.001; ns, nonsignificant vs. appropriate control.

These findings in cultured cells prompted us to investigate the effect of curcumin on Hp viability in mice and its regulation on MMPs in gastric tissues. To test the effect of curcumin on Hp-infected mice, two weeks AM1 or SS1 Hp infected mice were given curcumin treatment for 7 consecutive days and then sacrificed. Since curcumin has anti-infectious properties in cultured cells, we tested the comparative effect of curcumin with TT on Hp-colonization in mice. Quantitative-culture showed that curcumin eradicated Hp from infected mouse stomach and was equipotent to TT (Figure 3A). Urease test conducted with the respective mouse gastric tissues also confirmed the protective effect of curcumin (Figure 3A). It is noteworthy that curcumin added exogenously had no effect on Hp urease activity and thus the possibility of its interference with the urease test is remote. To further confirm curcumin's anti-Hp potential, Hp-specific genes *ureB* and *napA* were amplified by PCR using DNA isolated from mouse gastric tissues of Hp-infected with or without curcumin treated mice. Mouse specific GAPDH gene served as control. Figure 3B confirms active infection of Hp within 2 weeks and that curcumin or TT completely eliminated Hp from mouse stomach. These data indicate that even a dose of 25 mg/kg of curcumin was equipotent to the human dose of TT in eradicating Hp *in vivo*. The activities of secreted MMP-3 and -9 (Figure 3C and D) that escalated significantly due to Hp-infection was reduced dose dependently by curcumin treatment and reduced to control level by either 25 or 50 mg/kg b.w. while the TT treatment diminished the activity by only 50% at either doses used (Figure 3E and F). This significant difference in downregulation of elevated MMP-3 and -9 secretions by curcumin and TT led us to speculate a possible differential mechanism of protection between the two in protecting against Hp infection.

### Histological and immuno-histochemical analysis of Hp infected tissues and effect of curcumin

**Figure 4:**
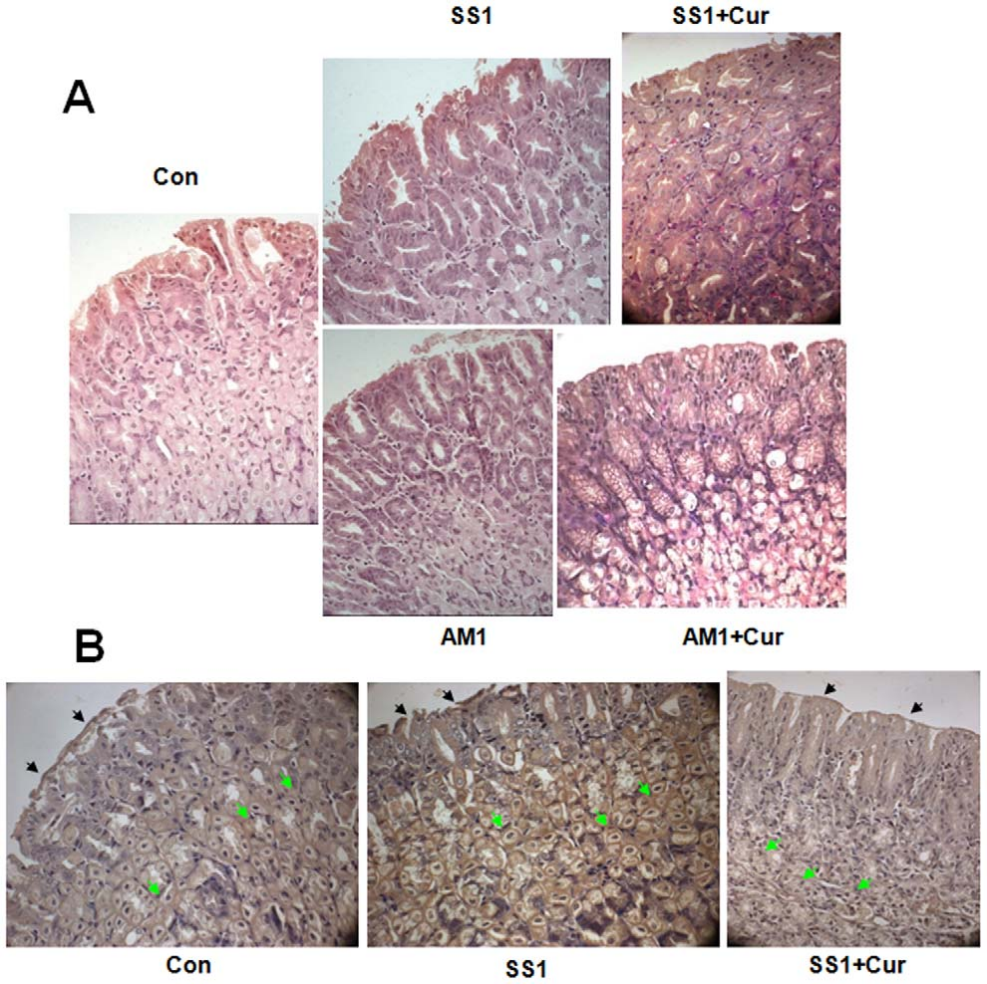
Histology and immunohistochemistry of mouse gastric tissues after Hp-infection and eradication; MMP-9 suppression by curcumin. Two weeks SS1 or AM1 strain of Hp infected mice were orally fed with 25 mg/kg b w curcumin for 7 consecutive days. Mice were sacrificed and antral biopsy specimens were processed for histological analysis. The expression of MMP-9 in gastric mucosa was analyzed by immunohistochemistry. Histological section of control, SS1 infected, AM1 infected, curcumin treated SS1 infected and curcumin treated AM1 infected tissues were stained with hematoxylin and eosin. Photographs were taken at 40× magnification respectively (**A**). Immunohistochemistry of control, SS1 infected and curcumin treated SS1 infected tissues probed with anti-MMP9 antibody (**B**). Epithelial layer and parietal cells were marked by black and green arrows respectively.

To rule out the possibility of a strain specific effect of curcumin on Hp, cag^−ve^ strain AM1 were introduced in the study. Histological studies were performed to examine the eradication of Hp infection by curcumin at tissue level and to observe the morphological changes. Histological analysis of antral biopsy specimens revealed that Hp infection (both SS1 and AM1 strain) caused mucous depletion, loss of continuity of surface epithelium along with distortion and erosion of surface epithelial cells at places compared with that of control. Presence of Hp onto the epithelial surface was visible for both SS1 and AM1 infected tissues and was confirmed by modified Giemsa stain. Epithelial damage was more pronounced in SS1 infected tissues compared to AM1 infected tissues (Figure 4A). This evidence of mucosal damage was completely abolished by curcumin treatment. Eradication of Hp as well as regaining of the normal contour of surface epithelium of antral mucosa was observed following curcumin treatment in both SS1 and AM1 infected tissues. This data emphasizes that curcumin is effective against Hp strains with different genetic background. Whether, Hp eradication was associated to MMP-9 mediated pathway, immunohistology was performed using anti-MMP-9 antibody. The localization of MMP-9 in most of the antral mucosal cells especially in the cytoplasmic part of parietal cells throughout the section was prominent in SS1 infected tissues (Figure 4B). However, the expression of MMP-9 was significantly reduced in almost diffused pattern throughout the antral mucosa due to curcumin treatment. Altogether, this confirms that during Hp infection the epithelial layer was eroded and damaged while curcumin was highly effective in suppressing MMP-9 and healing of overall damage caused by infection.

### Curcumin restricts MMP-3 and -9 during protection of cag^+ve^ and cag ^–ve^ Hp-infection in mice

**Figure 5:**
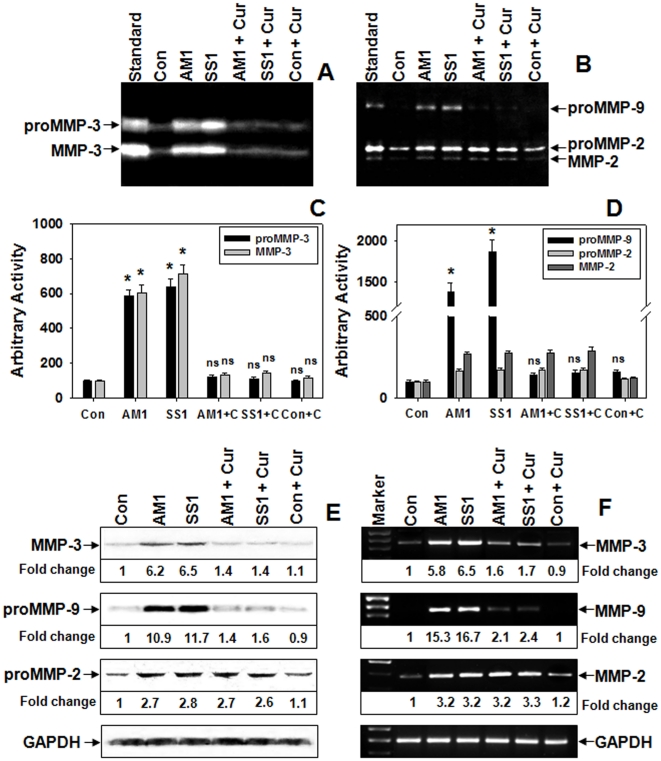
Curcumin regulates MMP-3 and -9 activity and expression in both cag^+ve^ and cag^−ve^ Hp-infected mice. Two weeks Hp (either SS1 or AM1) infected C57BL/6 mice were orally-fed with curcumin for 7-days. The activities of MMP-3, and MMP-9, -2 in the PBS extracts of respective mouse gastric tissues were analyzed by casein (**A**) and gelatin (**B**) zymograms respectively. Histographic representations of caseinolytic (**C**) and gelatinolytic (**D**) activities from the above zymograms and three other representative zymograms from independent experiments. Error bars = ±SEM. *, p<0.001; ns, nonsignificant vs. appropriate control. Western blot analysis of MMP-3, -9 and -2 proteins from respective tissues, GAPDH served as loading-control (**E**). RT-PCR analysis of MMP-3, -9 and -2 mRNA expressions in respective tissues while GAPDH served as control (**F**).

Since, curcumin was almost equally effective at 50 or 25 mg/kg b.w., lower dose (25 mg/kg b.w.) was used in further mice experiments. Differences in MMPs activity were negligible in SS1 and AM1 infected gastric tissues (Figure 5A and B). In infected mice the activities of secreted pro and active MMP-3 (Figure 5A and C) and proMMP-9 (Figure. 5B and D) that escalated due to Hp were reduced to almost control level by curcumin irrespective of Hp-strains. However curcumin's effect on MMP-2 activity was negligible (Figure 5B and D). Only curcumin administered mice showed no changes in MMPs activity and were similar to control. To analyze whether MMPs are regulated at the protein and mRNA levels, gastric tissue samples from Hp-infected and control with or without curcumin treatment were assessed by Western blot and RT-PCR. The negative regulation on MMP-3 and -9 activities by curcumin was also evident at the protein (Figure 5E) and mRNA levels (Figure 5F). A 6-fold increase in MMP-3 protein in both SS1 and AM1 infected tissues was inhibited to control level by curcumin. In addition, the MMP-9 expression increased ∼10 fold and curcumin reversed it very efficiently for both cag^+ve^ and cag^−ve^ strains, while MMP-2 expression remained unaltered (Figure 5E and F).

### Comparison between the potentials of curcumin and TT to regulate the altered balance of MMPs vs TIMPs during protection of Hp-infection

**Figure 6:**
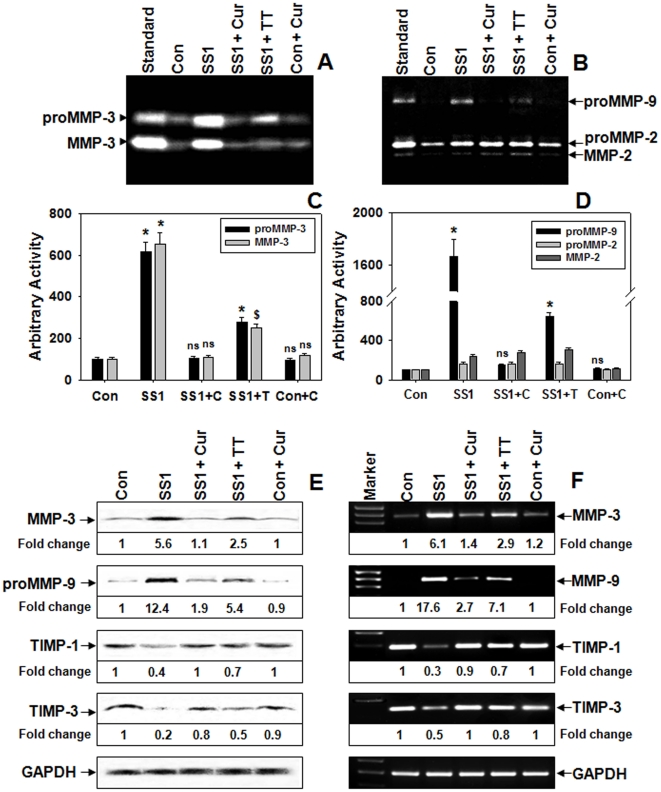
Comparison between curcumin and TT in modulating altered levels of MMPs and TIMPs during Hp-infection. Two weeks SS1 infected mice groups were treated with curcumin or TT for 7-days. The activities of MMP-3 and gelatinases in the PBS extracts of respective mouse gastric tissues were analyzed by casein (**A**) and gelatin (**B**) zymograms respectively. Histogram representing caseinolytic (**C**) and gelatinolytic (**D**) activities of the above zymograms and three other representative zymograms from independent experiments. Error bars = ±SEM. *, p<0.001; $, p<0.01; ns, nonsignificant vs. appropriate control. Western blot (**E**) analysis of protein expressions of MMP-3, -9, TIMP-1 and -3 in respective samples, GAPDH served as loading-control. RT-PCR (**F**) analysis of MMP-3, -9, TIMP-1 and -3 mRNA expressions in respective gastric tissues, GAPDH served as control.

Next we compared the efficiency of curcumin with that of TT in regulating the activities and expressions of MMP-3 and -9 in Hp-infected mice. Since curcumin was almost equally effective for both AM1 and SS1 strain infections, SS1 being cag^+ve^ was chosen as the representative strain for this study. Figure 6A and C documents that TT was able to downregulate Hp-induced secreted pro and active MMP-3 activities, however the effectively of curcumin was much more promising. The secretion of proMMP-9 in infected tissues diminished by ∼60% by TT compared to ∼90% by curcumin treatment (Figure 6B and D), while secreted pro and active MMP-2 was unaltered. Data from protein and mRNA confirmed that the enhanced secretion of MMPs was due to increased synthesis at protein lavel (Figure 6E and F). The expression of TIMP-1 that significantly declined at protein as well as mRNA levels due to Hp-infection was restored to almost control level by curcumin while TT was ∼60% effective at both levels (Figure 6E and F). Similar to TIMP-1 the expression of TIMP-3 protein, that was also reduced by Hp-infection was rescued to ∼80% and ∼50% of control value by curcumin and TT respectively (Figure 6E). The mRNA level of TIMP-3 that drifted significantly in Hp-infected tissues was reversed to almost control level by curcumin and halfway by TT (Figure 6F). Notably, no significant changes were observed between the MMP levels of control and curcumin-control samples indicating that curcumin alone at this particular dose had no detectable effects on gastric MMPs. These results not only elucidate the effect of TT on gastric MMPs but also confirm that curcumin is more effective than TT in restoring the altered balance between MMPs vs TIMPs in gastric mucosa during protection of Hp-infection. We reasoned that this differential downregulation of MMP-3 and -9 by curcumin and TT might be because the effect of the later on these MMPs was mainly due to Hp-removal while curcumin may have acted on the upstream signaling molecules involved in MMP regulation apart from Hp-eradication. To ascertain whether the effect of TT was solely due to Hp-eradication, only-antibiotics treated mouse-group was introduced in the study. Zymographic analysis revealed that the activities of MMP-3 and MMP-9 were almost identically reduced in TT or only-antibiotics treated mice (Figure S1A, B, C and D). Similar results were also observed at the levels of MMP-3 and -9 mRNA expression (Figure S1E) suggesting that their downregulation by TT was mainly due to Hp-eradication. To precisely determine the expression levels of MMP-3 and -9 in Hp-infected and curcumin or TT or only-antibiotics treated gastric tissues, quantitative real time-RT-PCR was conducted. q-PCR analysis (Figure S1F) demonstrated that Hp-induced increased expressions of MMP-3 and -9 were more effectively abated by curcumin than TT, while similar effect of TT and only-antibiotics further validated that TT mediated MMP regulation was largely due to Hp-eradication.

### Regulation of MMP-3 and -9 by curcumin via NF-kB-dependent mechanism during protection of Hp infection in mice

**Figure 7:**
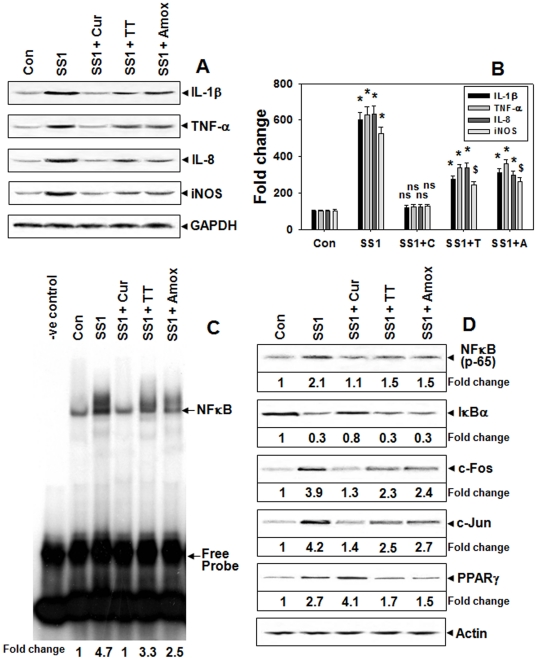
Regulation of MMP-3 and -9 via NF-kB dependent mechanism by curcumin-therapy in Hp-infected mice. Two weeks SS1-infected mice groups were treated with curcumin or TT or only-antibiotics for 7-days and the expressions of IL-1β, TNF-α, IL-8, and iNOS proteins were assessed by Western blot (**A**). Histographic representation of protein fold changes from the above blots and two other representative blots from independent experiments (**B**). Error bars = ±SEM. *, p<0.001; $, p<0.01; ns, nonsignificant vs. appropriate control. NFκB activation in the nuclear extracts of respective tissues using EMSA (**C**). Western blot analysis of NFκB(p65), IκBα and PPARγ proteins in Tx extracts and c-Fos and c-Jun proteins in the nuclear extracts of respective tissues while actin served as loading-control (**D**).

We next investigated the involvement of regulatory molecules possibly responsible for the differential downregulation of MMP-3 and -9 by curcumin-treatment and TT in Hp-infected mouse. Pro-inflammatory cytokines and iNOS are known to regulate MMP expressions directly or via transcription factors including NFκB and AP-1. The increased expressions of interleukin (IL)-1β, tumour necrosis factor (TNF)-α, IL-8 and iNOS during Hp-infection were better abrogated by curcumin-treatment than TT (Figure 7A and B). EMSA using nuclear extracts of mouse gastric tissues document that Hp-infection significantly activated NFκB (Figure 7C) that was blocked by curcumin to almost control level, while TT in contrast was less effective. Moreover, Hp-induced increased expression of NFκB (p65) and reduced IκBα protein level, the specific inhibitor of NFκB-activation [24], were more effectively restored by curcumin-treatment than TT (Figure 7D). The increased incidence of c-Fos and c-Jun, the key molecules in AP-1 complex [25], in the nuclear extracts of Hp-infected mouse gastric tissues were more effectively abrogated by curcumin than TT (Figure 7D). The expression of PPAR-γ, a NFκB and AP-1-activation antagonist [26]–[28], that increased during Hp-infection was surprisingly elevated even further by curcumin-treatment, while TT reduced its level indicating that their mode of action were distinctly different (Figure 7D). In the above experiments, results for only-antibiotics treatment was almost similar to that of TT confirming that bacterial eradication was the sole cause of their effect on these regulatory molecules. Altogether, these results suggest that curcumin in addition to Hp eradication, potentially targeted the regulatory molecules of MMP-3 and -9 in Hp-infected mice that probably lead to the more effective downregulation of these MMPs compared to TT.

## Discussion

Expression and secretion of different MMPs due to Hp-infection have been postulated to be critically involved in the development of gastric ulcer and gastric cancer [7], [8], [10]. However, recent evidences suggest that apart from its well studied inflammatory and pathogenic functions, MMPs play a more complex and diverse role in ECM homeostasis, regulation of inflammation, arresting disease progression and protecting cancer performing essential host functions [13], [29]–[31]. Since MMP-3 and -9 plays a pivotal role in gastric ECM degradation during Hp induced pathogenesis, and are associated with various carcinomas including gastric cancer [8], [12], attenuation of their increased secretion and synthesis is critical for restoration of the gastric damage caused by Hp infection. Although a majority of Hp strains do not colonize mouse, the few that do have substantially contributed to understand the pathogenic mechanism of this bacterium and helped largely in the development of therapeutic strategies against it. Since the C57BL/6 mouse model of Hp infection is robust and widely used to examine host responses to Hp-infections, antimicrobial therapies as well as Hp eradication studies, we tested the effect of curcumin and TT in this system. The primary requisites for good quality healing of Hp infection include eradication of the bacterium as well as restitution of the cellular and molecular changes inflicted by its infection. The reason behind using AGS cells was because several studies have shown that Hp interacts specifically with gastric epithelial cells, activating signaling pathways thus modifying host cellular functions [32], [33]. Moreover, in Hp infected mucosa, epithelial cells appear to be one of the major sources of MMPs [7], [8], [10], although MMPs are secreted from various cell types including macrophages, neutrophils and fibroblasts [12]. Though the potential of TT on Hp eradication has been rigorously studied, however its action on gastric MMPs has gained no attention. Thus, in this study we compared the efficiency of curcumin with that of TT in regulating gastric MMP-3 and -9 in Hp-infected mice.

Although curcumin has long been used as a dietary spice, it has recently been shown to possess antibacterial, antiulcerogenic and anticarcinogenic effects on various experimental models [9], [19], [20], [34]. Here, we tested the mechanism of curcumin's action on protection against Hp-infection in C57BL/6 mice. We demonstrate that curcumin, apart from eradication of Hp strains from infected mice, regulated the expressions and activities of MMP-3 and -9 in the gastric tissues. Moreover, curcumin was highly effective in restoring the denudation of epithelial region, disruption in gastric mucosal layer and infiltration of inflammatory cells that occurred due to Hp infection in mouse gastric tissues, as shown previously [23]. We also compared the effect of triple therapy on SS1 infected gastric tissues and found that curcumin was more effective in reducing the infiltration of inflammatory cells compared to TT. The effect of curcumin on gastric MMPs *in vivo* was consistent with its effect on Hp infected AGS cells. We show that TT mediated Hp eradication was also associated with moderate downregulation of MMP-3 and -9. It is known that repair of damaged tissues and remodeling of the ECM are governed by the balance between MMPs and TIMPs [12]. The altered balance between the MMPs and TIMPs during Hp-infection in mice was normalized more efficiently by curcumin than TT in protection studies. To clarify whether the effect of TT on these MMPs in infected tissues was solely due to Hp eradication, antibiotic alone was considered for comparative study. Our results demonstrate that TT or only-antibiotics almost similarly downregulated MMP-3 and -9 activities while curcumin was more effective during protection against Hp-infection in mice and cultured cells. The similarity in the outcome of TT and only-antibiotics treatments clearly indicated that the effect of TT on these MMPs and TIMPs was entirely because of Hp eradication while curcumin was effective in both Hp eradication and biochemical regulation of these MMPs that synergistically influenced good quality healing and tissue restitution. These findings raised an important question, whether curcumin may be considered as an alternative therapy against Hp-infection since TT has considerable complications and side effects on patients [4], [5].

An increasing pool of evidence suggests the increased expressions of several pro-inflammatory cytokines and iNOS in Hp-associated gastroduodenal disorders [7], [10], [35], [36]. Among them, TNF-α and IL-1β are essentially involved in the activation of NFκB in gastric epithelial cells [36], which in turn induces several inflammatory genes including IL-8, iNOS and several MMPs. Moreover, IL-8 has been reported to directly participate in MMP-9 induction [37]. Furthermore, iNOS synthesizes nitric oxide (NO), which has diffuse functions associated with infection, inflammation and carcinogenesis [38], [39]. We found a substantial increase in the expressions of TNF-α and IL-1β in Hp-infected mice that were effectively blocked by curcumin compared to TT. Reasonably, similar results were achieved for IL-8 and iNOS. Since NO has been indicated to increase the activities and expressions of MMPs including MMP-9 and downregulate TIMP-1 [40], [41], the NO generated by iNOS during Hp-infection might have a significant role in elevating the activities of MMPs and suppression of TIMPs. Collectively, these results suggest that curcumin in addition to Hp removal potentially targeted the pro-inflammatory cytokines and iNOS possibly because of its anti-inflammatory property which in turn might have directly or via NFκB or AP-1 signaling, stabilized the MMPs/TIMPs balance during tissue restitution following Hp-eradication. However, TT mediated changes in these cytokines and iNOS being almost identical to that of only-antibiotics indicate that Hp removal as the sole cause of this effect.

The promoters of MMP-3 and -9 carry putative NFκB and AP-1 binding sites and previous studies show that Hp induces MMPs via NFκB and AP-1 signaling [8], [12], [15], [42], [43]. Moreover, the elevated levels of proinflammatory cytokines and iNOS in Hp-infected mice as discussed previously were indicative of NFκB and AP-1 involvement in the process of MMP-3 and -9 upregulation. We found increased activation of NFκB in Hp-infected mice that were more effectively blocked by curcumin-treatment than TT. Additionally, curcumin blocked the degradation of IκBα more effectively than TT during protection of Hp-infection. The elevated nuclear abundance of both c-fos and c-jun proteins during Hp-infection *in vivo* was also better suppressed by curcumin-treatment than TT suggesting AP-1 inhibitory potential of curcumin. Our findings were consistent with previous reports indicating curcumin mediated MMP regulation through NFκB and AP-1 signaling pathways in other pathogenic models [44]–[46]. Reports indicate that transcription factor PPARγ inhibits the expression of several MMPs by antagonizing the activities of AP-1 and NFκB and has recently been considered as an important target for development of new drugs for cancer therapy [26]–[28], [47]. We found increased expression of PPARγ during Hp-infection in mice, in accordance with previous reports [48], which was reduced by TT, while curcumin-treatment on the contrary further elevated its level. We believe that Hp-mediated induction of PPARγ might be a feedback mechanism to partly suppress the exaggerated inflammation that may have occurred through NFκB and AP-1 and consequently perturbed the long-term survival of Hp in the host. While curcumin induced modulation of PPARγ might be a transient effect to counteract the Hp-induced activation of NFκB and AP-1 and, subsequent MMP-3 and -9 upregulation. This effect of curcumin on PPARγ was distinctly different from that of TT while treating Hp-infection possibly because curcumin exhibit PPARγ ligand-binding property and stimulates its expression and activity [49]. Altogether, increased expression of PPARγ and reduced degradation of IκBα led to organized downregulation of NFκB activity by curcumin but not by TT. On the contrary, TT moderately reduced the expression of NFκB p65 possibly due to Hp removal without altering PPARγ expression or IκBα degradation. Thus, our results conclusively indicate that curcumin mediated downregulation of MMP-3 and -9 in Hp-infected mice was via suppression of NFκB and AP-1 activation and activation of PPARγ. Our results suggest that curcumin acted both-ways during protection of Hp-infection by eradicating Hp as well as potentially targeting the key molecules (MMP-3 and -9) involved in the Hp-induced gastric diseases.

In conclusion, our study demonstrates that elevated levels of MMP-3 and -9 in gastric tissues of mice or cultured cells due to infection by Hp strains (either cag^+ve^ or cag^-ve^) are inhibited by curcumin treatment. Curcumin is more effective than TT in restabilizing the altered balance between MMPs and TIMPs during protection against Hp-infection. This curcumin mediated downregulation of MMP-3 and -9 levels in Hp-infected mice and cultured cells suggest its immense therapeutic potential against Hp associated gastrointestinal diseases. This study also documents the potential mechanism of action of TT on these MMPs and their regulators in mouse gastric tissues during Hp removal. Our study highlights the potential of curcumin-based therapy as a promising anti-Hp agent having property to restore and repair the gastric damage caused by Hp-infection. Since curcumin is cheap and easily available in developing countries like India, this study opens scope for an easy therapeutic solution to a potentially complicated Hp-related disease.

## Methods

### Ethics Statement

This study was carried out in strict accordance with the guidelines of Council of Scientific and Industrial Research, Govt of India. The protocol was approved by the Animal Ethics Committee of Indian Institute of Chemical Biology (Permit Number: 147/1999 CPCSEA) affiliated to Indian Institute of Chemical Biology (a unit of Council of Scientific and Industrial Research), Kolkata. All experiments were performed under standard controlled conditions and all efforts were made to minimize animal suffering.

### Hp strains and culture

Mouse-adapted Hp strains SS1 and AM1, were grown on brain-heart infusion agar as described earlier [10]. Nalidixic acid (10 µg/ml), polymyxin B (10 µg/ml) and bacitracin (200 µg/ml) were added to this medium when culturing Hp from mouse stomachs and cultured cells. The plates were incubated at 37°C under 5% O_2_, 10% CO_2_, 85% N_2_. In all the experiments, overnight grown cultures were used. The minimum inhibitory concentration (MIC) for curcumin was determined by standard agar dilution method. Final test concentrations consisted of 100, 50, 20, 15, 10, 5 µg/ml curcumin (Sigma Chemical Co, St. Louis, MO, USA), vehicle solvent served as control. The plates were inoculated with a bacterial suspension (10^8^ cfu/ml) in sterile phosphate buffered saline (PBS).

### Hp infection in C57BL/6 mice and treatment with curcumin

Specific pathogen free C57BL/6 mice bred in house were used in all experiments. Experiments were designed to minimize animal suffering and to use the minimum number associated with valid statistical evaluation following the guidelines of animal ethics committee of the institute. Animals were anesthetized by ketamine (12 mg/kg b.w.) followed by cervical dislocation for killing. Animals of both control and experimental groups were kept separately in standard conditions and fasted for 6 h with free access to water before each inoculation. Groups of mice (12 mice/group) were inoculated with SS1 or AM1 or PBS twice in a period of three days with ∼10^8^ cfu/mouse/inoculation as depicted previously [10]. After 2-weeks from final inoculation the mice were orally-fed with curcumin (25 mg/kg or 50 mg/kg b.w.) (Sigma Chemical Co, MO, USA) or triple-therapy (omeprazole, tinidazole and amoxicillin) or only-antibiotics (tinidazole and amoxicillin) (HP-Kit, Sun Pharmaceuticals. India) (0.0013 or 0.0026 times human-dose) [50], for 7-days consecutively, while untreated ones received sterile water and curcumin control group received only curcumin.

### Cell culture and Hp infection

The human gastric epithelial cells (AGS) maintained as described earlier [10], were transferred into six-well tissue-culture plates 24 h before infection. For coculture experiments, Hp strains were harvested in PBS, centrifuged, resuspended in antibiotics/FBS-free media at 1×10^9^ cfu/ml concentration and immediately incubated with AGS cells at a bacteria/cell concentration of 100∶1. Various doses of curcumin and 8.9×10^−2^ fold/mouse-dose (ratio between µg of curcumin/mouse to 60 µM curcumin/well) of TT, only-antibiotics or omeprazole were administered to AGS cells/well 30 min prior Hp-infection and cultured in antibiotics/FBS-free media for 24 h. Media was concentrated 10-fold by lyophilization to use for gelatin zymography and Western blot.

Urease-test. Urease-test was conducted using small portions of mouse gastric tissues as described by Chattopadhyay *et al* [51].

### DNA methods

Chromosomal DNA from mouse gastric tissues was extracted as depicted earlier [52]. The presence of specific bacterial genes and the specificity for mouse-genome was scored by PCR using specific primers (supplementary Table-1A) and DNA from respective tissues. PCR was carried out in 20μl reaction volumes using 10 pmoles of each primer, 0.25 mM of each dNTP, 1U of Taq polymerase (Invitrogen, CA, USA) and 40 ng of DNA for 40 cycles of denaturation (94°C for 30 s), primer-template annealing (57°C for 30 s), and DNA synthesis (72°C for 1 min). The PCR-products were analyzed by electrophoresis in 2% agarose-gels, product sizes were estimated by 100 bp or 50 bp-markers (Invitrogen).

### Tissue extraction and partial purification of gelatinases

The body and the pyloric parts of mouse stomach were suspended in PBS containing protease-inhibitors (Sigma), minced, centrifuged at 12,000 *g* for 15 min and the supernatant was collected as PBS extracts, while the pellet was re-extracted in lysis-buffer to obtain Tx extracts [10]. A portion of the stomach was minced in PBS and used for quantitative-culture to score bacterial colonization. For partial purification of MMP-9 and -2, PBS extracts of respective samples were incubated with gelatin-agarose beads, at 4°C for 1 h followed by centrifugation at 1500 *g*. The pellet was washed twice with PBS through centrifugation at 1500 *g* and the gelatinases were eluted in Lammeli sample loading-buffer [53].

### Gelatin and casein zymography

For assay of MMP-3 activity, casein zymography and for assay of MMP-9 and -2 activities, gelatin zymography were performed as described previously [10]. Standard MMP-9 and MMP-2 enzymes were purchased from Chemicon, Hampshire, UK. Hp-infected human gastric tissue extract was used as MMP-3 standard. Quantification of zymographic-bands was done using Lab-Image software.

### Western blotting

Tissue extracts (100 µg/lane) were subjected to Western blotting as described earlier [10]. Polyclonal-mouse-reactive anti-MMP-3, anti-MMP-9, anti-MMP-2, anti-TIMP-1, anti-TIMP-3, anti-IL-1β, anti-IL-8, anti-TNF-α, anti-iNOS, anti-NFκB(p65), anti-IκBα, anti-PPARγ, anti-c-Fos, anti-c-Jun, anti-GAPDH and monoclonal-human-reactive anti-MMP-9 antibodies were purchased from Santa Cruz Biotechnology, Santa Cruz, USA. Western blots shown in each case are representative blots from at least three independent experiments.

### Reverse transcriptase-PCR (RT-PCR)

Total cellular RNA extraction and complementary-DNA synthesis were done as described previously [10]. The cDNA (1 µl) was amplified in 20 µl reaction buffer for 35 cycles of denaturation (94°C for 30 s), annealing (58°C for 30 s), and extension (72°C for 1 min) using specific primers (Table-1B). The PCR-products were electrophoresed in 2% agarose-gels and product sizes were estimated by 100 bp-marker.

**Table 1:** 

A. Details of PCR primers used for analysis of *ure*B, *nap*A and mouse GAPDH genes in DNA isolated from mouse gastric tissues.			
Locus name	Primer pairs	Sequence	Ampliconsize (bp)
*ureB*	ureB-F	5′-CGTCCGGCAATAGCTGCCATAGT-3′	464
	ureB-R	5′-GTAGGTCCTGCTACTGAAGCCTTA-3′	
*napA*	napA-F	5′-TCCTTTCAGCGAGATCGTCA-3′	95
	napA-R	5′-GAATGTGAAAGGCACCGATT-3′	
*GAPDH*	GAPDH-F	5′- GCAGTGGCAAAGTGGAGATT -3′	249
(mouse)	GAPDH-R	5′- TCTCCATGGTGGTGAAGACA -3′	

B. Details of PCR primers used for mRNA expression analysis of mouse gastric tissues.			
mRNA name	Primer pairs	Sequence	Ampliconsize (bp)
*MMP-3*	MMP-3-F	5′- GGATTGTGAATTATACACCGGAT -3′	769
	MMP-3-R	5′- GGATAACCTGCTAGCTCCTCGT -3′	
*MMP-9*	MMP-9-F	5′- GACCCGAAGCGGACATTGTCAT -3′	755
	MMP-9-R	5′- GGAATGATCTAAGCCCAGTGCAT -3′	
*MMP-2*	MMP-2-F	5′- ATGGCTTCCTCTGGTGCTC -3′	385
	MMP-2-R	5′- TCGTAGTGGTTGTGGTTGC -3′	
*TIMP-1*	TIMP-1-F	5′-CCTCTGGCATCCTCTTGTTG-3′	285
	TIMP-1-R	5′-TGCGGTTCTGGGACTTGT-3′	
*TIMP-3*	TIMP-3-F	5′-TTGGGTACCCTGGCTATCAG-3′	132
	TIMP-3-R	5′-AGGTCTGGGTTCAGGGGTCT-3′	
*GAPDH*	GAPDH-F	5′- TGGGGTGATGCTGGTGCTGAG -3′	497
	GAPDH-R	5′- GGTTTCTCCAGGCGGCATGTC -3′	

### Real time-RT-PCR

The real time-RT-PCR was carried out in a 20 µl volume containing 50 ng cDNA, 10 pmoles of each primer and SYBR green PCR-master mix with Real-Time PCR System 7300 (Applied Biosystems, CA, USA). Polymerase activation at 95°C for 5 min followed by 55 cycles at 94°C for 30 s, 58°C for 30 s and 72°C for 1 min. A quantitative measure of MMP-9/-3 was obtained through amplification of GAPDH and MMP-9/-3 cDNA in each sample. The amount of MMP-9/-3 expressions relative to the total amount of cDNA was calculated as ΔCt = Ct_GAPDH_ – Ct_MMP-9/MMP-3_, where Ct_MMP-9,_ Ct_MMP-3_ and Ct_GAPDH_ were fractional cycle number at which fluorescence generated by reporter dye exceeded fixed level above baseline for MMP-9, -3 and GAPDH cDNA respectively. The changes of MMP-9/-3 expressions in respective samples compared to control were expressed as ΔΔCt = ΔCt_control_ – ΔCt_respective samples_. Relative expressions in MMP-9/-3 genes in respective samples were calculated as 2^ΔΔCt^. Each sample was run thrice.

### Electrophoretic mobility shift assay (EMSA)

EMSA was performed as previously described with a few modifications [54]. Briefly, mouse gastric tissues were minced, hand-homogenized and centrifuged at 1000 *g* for 5 min at 4°C. After washing with ice cold PBS, cell pellets were suspended in 200 µl low-salt buffer (10 mM HEPES pH-7.9, 1.5 mM MgCl_2_ and 10 mM KCl), incubated for 10 min on ice, followed by vigorous mixing after addition of 20 µl of 10% NP-40. Nuclei were collected by centrifugation and resuspending in 50 µl high-salt buffer (20 mM HEPES pH-7.9, 420 mM NaCl, 1.5 mM MgCl_2_, 0.2 mM EDTA, 25% glycerol). Both buffers were supplemented with protease inhibitors and 0.5 mM DTT. Nuclei were incubated for 15 min on ice, vortexed periodically and centrifuged at 12500 *g* for 10 min to obtain the nuclear extracts. NFκB-specific oliginucleotides 5′-AGTTGAGGGGACTTTCCCAGGC-3′ (sense), and 5′-GCCTGGGAAAGTCCCCTCAACT-3′ (antisense) were used for EMSA. Binding reactions were performed for 30 min on ice with 50 µg nuclear extract and (γ-^32^P) ATP labeled oliginucleotide. Binding complexes were electrophoresed in 7% nonreducing polyacrylamide-gels, dried and radioactive signals were detected by autoradiography.

### Histology

The body and the pyloric parts of control and two weeks infected and curcumin treated infected mouse stomach were sectioned for histological studies. The tissue samples were fixed in 10% formalin and embedded in paraffin. The sections (5 µm) were cut using microtome, stained with hematoxylin and eosin [10], and observed under an Olympus microscope. Images were captured using Camedia software (E-20P 5.0 Megapixel) at original magnification 40×10 processed in Adobe Photoshop version 7.0.

### Immunohistochemistry

Serial sections were deparaffinized in xylene and dehydrated through a graded ethanol series. For better detection, sections were pretreated with 0.03% trypsin for 1 hour at 37°C. Then the tissues were placed in 3% hydrogen peroxide and absolute methanol for 5 minutes to reduce endogenous peroxidase activity, followed by washing in PBS. The tissue sections were incubated with anti-mouse MMP-9 antibody (diluted 1∶200) or a control immunoglobulin G for 3 hours at 37°C. After washing with PBS, sections were covered with EnVision plus for 40 minutes at 37°C and washed in PBS. Antigenic sites bound by antibody were identified by reacting these sections with a mixture of 0.05% 3,3′-diaminobenzidine tetrahydrochloride in 50 mmol/L Tris-HCl buffer and 0.01% hydrogen peroxide for 7 minutes [8]. Sections were then hydrated in ethanol, cleaned in xylene, and mounted.

### Statistical analysis

Densitometry data were fitted using Sigma plot. Data were presented as the mean± SEM. Statistical analysis was performed using Student-Newman-Keuls test (ANOVA).

## Supporting Information

Figure S1 **Curcumin downregulates increased MMPs in cag^+ve^ Hp-infected mice more efficiently than either TT or antibiotics.** Two weeks SS1 infected mice groups were treated with curcumin or TT or only-antibiotics for 7-days. The activities of MMP-3 and gelatinases in the PBS extracts of respective mouse gastric tissues were analyzed by casein (**A**) and gelatin (**B**) zymograms respectively. Histographic representations of caseinolytic (**C**) and gelatinolytic (**D**) activities from the above zymograms and three other representative zymograms. Error bars = ±SEM. *, p<0.001; $, p<0.01; ns, nonsignificant vs. appropriate control. RT-PCR (**E**) analysis of MMP-3 and -9 mRNA expressions in respective gastric tissues, GAPDH served as control. (**F**) Histographic representation of relative expressions of MMP-9 and -3 transcripts in SS1-infected, curcumin or TT or only antibiotics treated mouse gastric tissues as measured by real time-RT-PCR. Error bars = ±SEM. *, p<0.001; $, p<0.01, vs. SS1+C.
(TIF)
