# Supplementary figures and images for: Curcumin Alleviates Matrix Metalloproteinase-3 and -9 Activities during Eradication of Helicobacter pylori Infection in Cultured Cells and Mice
Source: PLoS One. 2011 Jan 21;6(1):e16306. doi: 10.1371/journal.pone.0016306 (PMC3025008; doi:10.1371/journal.pone.0016306)

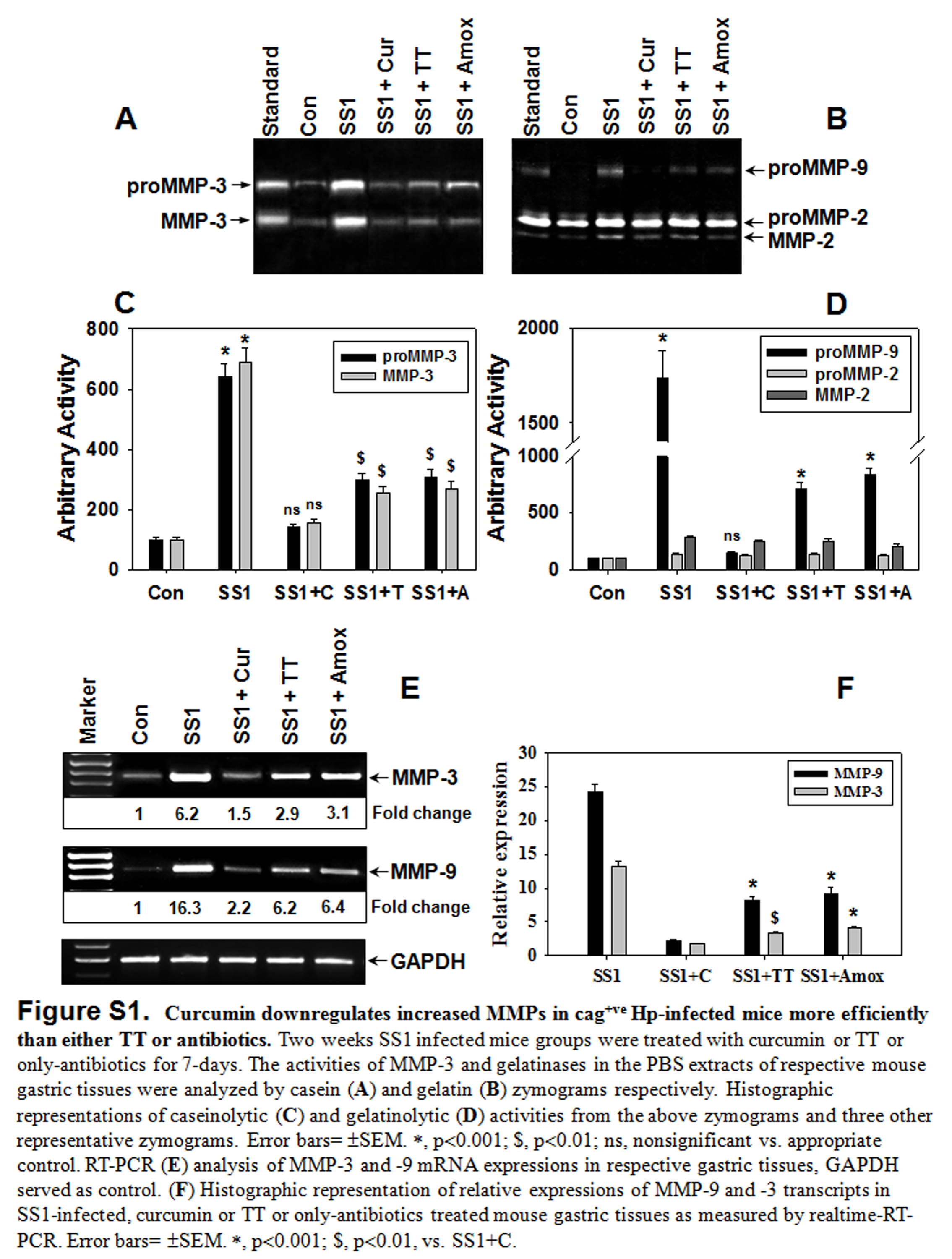

Supplement: Figure S1 — Curcumin downregulates increased MMPs in cag+ve Hp-infected mice more efficiently than either TT or antibiotics. Two weeks SS1 infected mice groups were treated with curcumin or TT or only-antibiotics for 7-days. The activities of MMP-3 and gelatinases in the PBS extracts of respective mouse gastric tissues were analyzed by casein (A) and gelatin (B) zymograms respectively. Histographic representations of caseinolytic (C) and gelatinolytic (D) activities from the above zymograms and three other representative zymograms. Error bars = ±SEM. *, p<0.001; $, p<0.01; ns, nonsignificant vs. appropriate control. RT-PCR (E) analysis of MMP-3 and -9 mRNA expressions in respective gastric tissues, GAPDH served as control. (F) Histographic representation of relative expressions of MMP-9 and -3 transcripts in SS1-infected, curcumin or TT or only antibiotics treated mouse gastric tissues as measured by real time-RT-PCR. Error bars = ±SEM. *, p<0.001; $, p<0.01, vs. SS1+C. (TIF) [file pone.0016306.s001.tif]
